# On to Nashville

**Published:** 1897-07

**Authors:** 


### ON TO NASHVILLE.

BUT one more issue of the JOURNAL will reach our readers before many of them will be on their way to Nashville. The programme is sufficiently well developed to assure all who may attend a feast of professional good things that shall enrich them a thousandfold. Every detail for our comfort is being planned by the local Committee of Arrangement. Hotel accommodations have been provided for, meeting-rooms obtained, and the hospitable doors of Nashville swing outward for our entrance. We have many valued members of our Association in the South, and there are many excellent members of the profession in that sunny region outside of our roll of members. They have all, in a greater or lesser measure, been pioneers in their calling in that rapidly developing section of our country. They are worthy of our support, our assistance, and our best thoughts, and the JOURNAL sincerely asks for the greatest outpouring of our members that we have seen for years. Let us go down among these fellow-colleagues and warmly second and aid every effort upon their part. Let us enroll in this region a larger number of these worthy representatives that we may more fully merit the name of "National" which we represent.

Let every State, city, and local association of veterinarians send duly accredited delegates, and every school, station, or branch of work of our calling be represented in this important gathering of our members, which will there deliberate and plan for the wiser and better advancement of our calling.

Our Secretary is tireless in his efforts to make Nashville a second to Buffalo, and everyone can strengthen his hands by urging the largest attendance; by suggestions as to the programme; by mingling in discussion with our brethren from all over this land, and thus accomplish the first great result of our efforts—higher education.

